# Notes and Comments

**Published:** 1898-08

**Authors:** 


### Notes and Comments.[1]

The Transmission of Acquired Characteristics.—At a recent meeting of the Academy of Stomatology, Professor C. N. Peirce gave a lecture on comparative odontology. He stated that his hearers probably appreciated the fact that he was a firm believer in evolution, an important factor in which is the tendency to variation. Variability in the development of all living beings is probably

[1] The assistant editor solicits contributions for this department,—new methods, new remedies and formulas, or any short practical note which may prove of value to the practitioner or student. Address 1338 Walnut Street, Philadelphia.

an unyielding law of nature, and the question in which we are all interested is how certain forms become fixed. In other words, what is the force which establishes the shape and size of organs? A theory advanced by Lamark, which has long been accepted by many scientists, is that the long-continued need of a structure begot a movement which was originated and encouraged by the long-felt want, and in turn played its part in the development of the needed organ. Also that the force and efficiency of an organ was in the ratio of the use, exercise, or function of the organ. Thus the doctor attempted, by the exhibition of diagrams and natural specimens of jaws and teeth, to illustrate the correctness of this theory, explaining that the shape of the teeth and the infoldings of the enamel were due to the movement of the jaw and the force and impact upon the structures, these being the result of the food habit. And further, that these changes, when acquired, must be transmitted, or the theory of evolution would not be sustained.

---

Wave-Length of the Röntgen Rays.—It is stated that Dr. Fromm, of Munich, has determined the wave-length of the Röntgen rays to be fourteen-millionths of a millimetre, or about seventy-five times smaller than the smallest wave-length of light. It has also been found that the alkaline metals are the most transparent to the Röntgen rays; and according to Professor Marangoni, lithium is the most transparent of these. Sodium is more transparent than potassium, a fact which may indicate that transparency is proportional to the weight of the atom and the density of the metal.

---

Student Societies.—Organizations among the students of our schools are rapidly increasing. They have proved of such value to the student's life that, in the dental department of the University of Pennsylvania, the Philadelphia Dental College, and the Pennsylvania College of Dental Surgery, there are at least eight active societies. This is another evidence of higher dental education, as the formation of debating societies must be of incalculable benefit in the forming of correct methods of managing such an organization and of presenting and discussing dental subjects.

---

Replantation for Child.—*The American Dental Weekly* reports a successful operation upon a girl, thirteen years of age, who, while walking in her sleep, fell upon a chair, which knocked out the left superior lateral incisor and cuspid. Four days afterwards she presented herself with the teeth in a bottle. The canal contents were removed, canals filled with gutta-percha, the ends of the roots were removed, the teeth were placed in warm carbolized water and then into formalin, the alveoli were cleansed, and the teeth replaced.

